# An Enhanced Mask R-CNN Approach for Pulmonary Embolism Detection and Segmentation

**DOI:** 10.3390/diagnostics14111102

**Published:** 2024-05-26

**Authors:** Kâmil Doğan, Turab Selçuk, Ahmet Alkan

**Affiliations:** 1 Department of Radiology, Kahramanmaras Sutcu Imam University, 46050 Onikişubat, Turkey; kamildogan@ksu.edu.tr; 2 Department of Electrical and Electronics Engineering, Kahramanmaras Sutcu Imam University, 46050 Onikişubat, Turkey; aalkan@ksu.edu.tr

**Keywords:** pulmonary embolism, Mask R-CNN, CTPA images

## Abstract

Pulmonary embolism (PE) refers to the occlusion of pulmonary arteries by blood clots, posing a mortality risk of approximately 30%. The detection of pulmonary embolism within segmental arteries presents greater challenges compared with larger arteries and is frequently overlooked. In this study, we developed a computational method to automatically identify pulmonary embolism within segmental arteries using computed tomography (CT) images. The system architecture incorporates an enhanced Mask R-CNN deep neural network trained on PE-containing images. This network accurately localizes pulmonary embolisms in CT images and effectively delineates their boundaries. This study involved creating a local data set and evaluating the model predictions against pulmonary embolisms manually identified by expert radiologists. The sensitivity, specificity, accuracy, Dice coefficient, and Jaccard index values were obtained as 96.2%, 93.4%, 96.%, 0.95, and 0.89, respectively. The enhanced Mask R-CNN model outperformed the traditional Mask R-CNN and U-Net models. This study underscores the influence of Mask R-CNN’s loss function on model performance, providing a basis for the potential improvement of Mask R-CNN models for object detection and segmentation tasks in CT images.

## 1. Introduction

**Figure 1:**
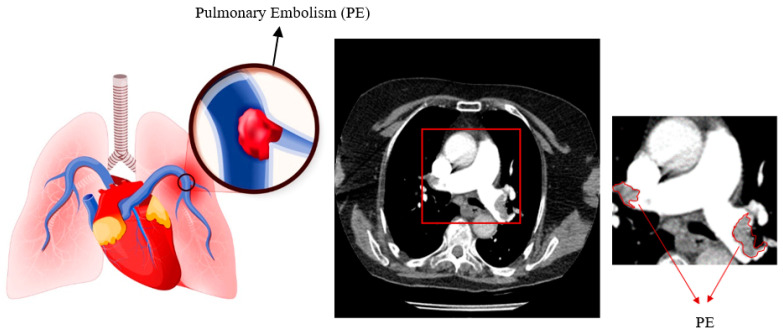
Representation of pulmonary embolism.

Pulmonary embolism (PE) is the obstruction of blood arteries in the lungs caused by a blood clot [1]. Peripheral edema has the third-highest prevalence among cardiovascular illnesses. The disease has a death rate of 30% [2,3,4]. A delay in diagnosing the condition increases the likelihood of impairment and mortality [5]. Early diagnosis is crucial to treating the disease effectively [6,7], with computed tomography pulmonary angiogram (CTPA) being the preferred method for diagnosing PE due to its quick and detailed imaging capabilities [8,9]. Blood arteries appear bright in contrast-enhanced CT scans due to the contrast material, while the embolism appears dark because it does not absorb the contrast agent. Figure 1 displays pulmonary embolisms in a high-quality computed tomography scan.

The detection of PE in CTPA images is performed manually by experienced radiologists; therefore, it can be time-consuming and sometimes difficult [10]. Some studies have shown that there is a 13% discrepancy between overnight and daytime assessments for the detection of PE [11,12,13]. In addition, in some emergency situations, the rapid and accurate assessment of PE is of great importance [14]. Some semi-quantitative methods can be used to measure the degree of vascular occlusion and determine the severity of PE. The most common methods for this purpose are the Mastora score and the Qanadli score, also known as the Vascular Obstruction Index (VOI), which are measured by an expert [15,16]. However, there is inconsistency among different experts regarding the use of these methods [17]. Therefore, researchers have considered the use of computer-assisted systems to automatically detect PE.

With the development of technological infrastructure, the PE detection performances of various algorithms have been increasing. Early studies on pulmonary fixation included limited clinical applications and showed poor performance. In these studies, clinical findings were used instead of CT images as reference materials. Initially, feature extraction was performed using simple artificial neural networks [18,19,20]; however, new algorithms have been developed for PE detection over time [21,22]. These studies have shown that computer-aided systems are successful in detecting PEs. It has also been shown that these systems can accurately detect small PEs, which may escape the eye of the specialist [22,23]. In a study, PE detection was performed automatically by classifying the features of PE using the *k*-Nearest Neighbors (*k*NN), artificial neural network (ANN), and Support Vector Machine (SVM) algorithms, and a sensitivity of 98% was obtained [24]. Machine learning algorithms have been widely used in recent years and have achieved high performances. There are many studies in which MR and CT images have been analyzed with deep learning algorithms, where much higher performances have been obtained [25,26,27]. Through the use of convolutional neural networks, high PE detection performance has been achieved in CTPA images [28,29]. Pham et al. combined natural language processing and machine learning for the diagnosis of thromboembolic disease [30]. CT-based deep learning and automated PE studies have distinct challenges when compared with their counterparts focusing on embolism in other locations. For example, PE data represent only a small fraction of images, compared with the size of the baseline CT data. There are also signal-to-noise problems when the intravenous contrast injection protocol and the patient breath-hold instructions are not followed [31,32]. For this reason, the correct design and creation of the neural network model and data set used have a great effect on performance. In particular, it is of great importance that the ground truth in the data set is created correctly. In recent years, significant progress in pulmonary embolism (PE) detection has been achieved through the development of novel algorithms and models. Grenier et al. developed the Hybrid 3D/2D U-Net model, which integrates 3D and 2D deep learning techniques tailored for CT image analysis. This model offers advanced segmentation capabilities to accurately identify pulmonary embolism regions [33]. Wu et al. explored computer-aided PE detection using VoxelNet, a deep learning method designed for the analysis of 3D CT images through processing volumetric elements (voxels) in order to automatically detect pulmonary embolisms and other airway obstructions [34]. Furthermore, Khan et al. achieved an 88% accuracy with a CNN model based on DenseNet201 when analyzing 9446 CT angiography scans from the RSNA-Kaggle database [35]. Additionally, Vainio et al. employed transfer learning and Maximum Intensity Projection (MIP) on images from the RSPECT data set to detect chronic PE [36]. These studies highlight the effectiveness of deep learning techniques in improving the detection and diagnosis of pulmonary embolism.

In this study, an enhanced mask R-CNN method is established for the detection and localization of pulmonary embolism in CT images, and the relationship between the loss function and the performance of the Mask R-CNN algorithm is determined. The results obtained show that the coefficients in the loss function significantly affect the performance of Mask R-CNN. For this study, a local data set containing masks for pulmonary embolism was also created. Details of the proposed method are given in the second part of this article. The results are presented in the third section. The last section includes our conclusions.

## 2. Materials and Methods

### 2.1. Materials

The data set was obtained from the Radiology Department of Kahramanmaraş Sutcu Imam University. The computed tomography images of 50 patients (27 of whom were female and 23 male) were used. The age range of the patients was between 28 and 95. The images were obtained with a Toshiba Aquilion ONE 320/640 Slice instrument. A raw data set with a total of 430 1212 × 1212 images in .tif format was created, which only included PE-containing sections. Furthermore, the sections in which a PE was displayed as the largest surface area were used. The CT scans were 8-bit (0–255) gray-level images. An average of 9 PE-containing sections were taken from each patient (min = 8; max = 12). An ethics committee report was obtained for the data set. All of the PEs in the images in this data set were labeled by one of the authors of this study (an expert radiologist with 15 years of experience) using the MATLAB ImageLabeler toolbox. In this study, only patients with pulmonary embolism (PE) and no other structures, such as lymph nodes, were included. Therefore, images were sourced from a limited pool of patients (50). Data augmentation techniques were applied to these images, including methods such as image rotation, contrast adjustment, vertical and horizontal flipping, and zooming, which are commonly used for data set enhancement. Specifically, horizontal and vertical rotations (±90°) and mirroring were employed, resulting in a fivefold increase in the number of images. As a result, 1630 images containing PEs were obtained. A total of 130 images (12 patients) were used for testing, and 1500 images (38 patients) were used for training.

### 2.2. Pre-Processing

**Figure 2:**
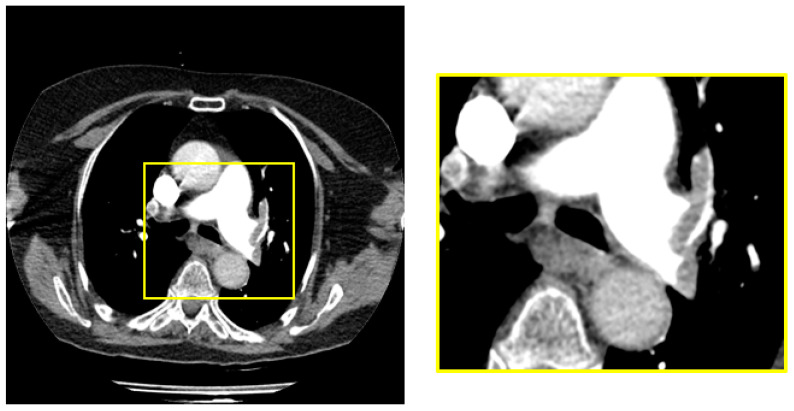
Obtaining the middle region of the image containing PE.

The dimensions of the raw images in the data set were 1212 × 1212. PEs were located in the middle region of all images in the raw data set. We obtained a 448 × 448 sub-image from the middle region of each raw image to cover the sections with pulmonary embolisms. Figure 2 shows a raw image and sample sub-image with PE.

### 2.3. Mask R-CNN Architecture

**Figure 3:**
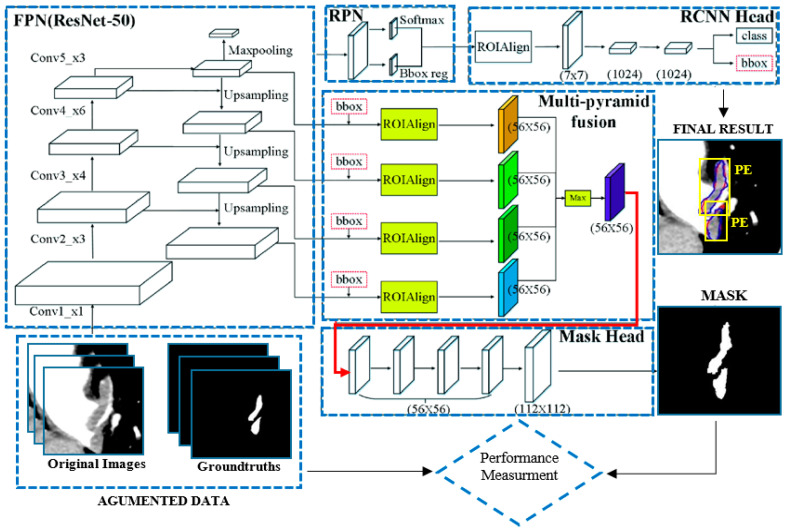
Mask R-CNN architecture [35].

The region-based convolutional neural network (R-CNN) [37], Faster R-CNN [38], and Mask R-CNN [39] approaches have been shown to possess high object detection performance. Unlike the other models, Mask R-CNN performs both detection and segmentation. This network is the extended version of Faster R-CNN, as it includes an extra segmentation pattern (i.e., segmentation mask). There are two phases in Mask R-CNN: in the first stage, feature extraction is performed for the regions; in the second stage, bounding-box detection, class detection, and segmentation are performed according to the extracted features. The Mask R-CNN architecture, which also includes the ResNet50 backbone network [40], is shown in Figure 3.

As can be seen in Figure 3, Mask R-CNN includes a Feature Pyramid Network (FPN) that allows for deep feature extraction. The FPN, designed according to the pyramid concept, is a network structure that excels in speed as well as accuracy. It has a multi-scale feature map and an upstream–downstream network structure. The upstream path is a convolutional neural network for feature extraction; as the number of upstream layers increases, the semantic value increases and high-level structures are detected. Regarding the backbone, as ResNet has a multi-layer structure, its training speed and estimation performance are quite high. In the basic structure of ResNet, there are hops between the front and back layers to facilitate back-propagation in the deep network training process. After the FPN in the proposed model architecture, there is an RPN, a deep convolutional neural network, which allows regions containing possible objects to be detected. The RPN takes data of any size as an input and proposes bounding boxes based on the object score. It makes this suggestion by shifting a small mesh over the feature map generated by the convolutional layer. After the RPN network, there is ROIAlign (i.e., an ROI alignment layer). This layer performs the same process as ROI pooling but solves the problem of unnecessary offsets in segmentation problems through the use of bilinear interpolation, thus achieving results much faster. This layer re-scales the image dimensions, which are then transmitted to the fully connected layer. Finally, the class and bounding box information for each region are obtained.

### 2.4. Enhanced Mask R-CNN Architecture

In this study, the Mask R-CNN structure was modified and the developed mask was trained as an R-CNN. In order to improve the pulmonary embolism detection performance, two weighting parameters, *λ*_1_ and *λ*_2_, were used in the loss function, which, in turn, consisted of the sum of classification loss, positioning loss, and segmentation loss, as shown in Equation (1).

(1) $$L=L_{cls}+{\lambda_{1}L}_{box}+{\lambda_{2}L}_{mask},$$

where *L_cls_* corresponds to the level at which the classes are detected incorrectly. In multi-object detection, this value should be increased if more than one object is not detected. *L_box_* is an adjustable parameter for the correct determination of the object’s boundaries, which can be increased if the bounding box is incorrectly positioned when the object is detected. On the other hand, *λ*_1_ and *λ*_2_ weigh the segmentation positioning losses of the object such that the network performance can be enhanced. *L_mask_* indicates how accurately the object is segmented and is expressed as follows:

(2) $$L_{mask}=-\frac{1}{m^{2}}\sum_{1\leq i,j\leq m}\left[y_{ij}{\log\hat{y}}_{ij}^{k}+\left(1-y_{ij}\right)y_{ij}{\log(1-\hat{y}}_{ij}^{k})\right],$$

where *y* and *ŷ* denote the label and predicted value, respectively.

### 2.5. U-Net

**Figure 4:**
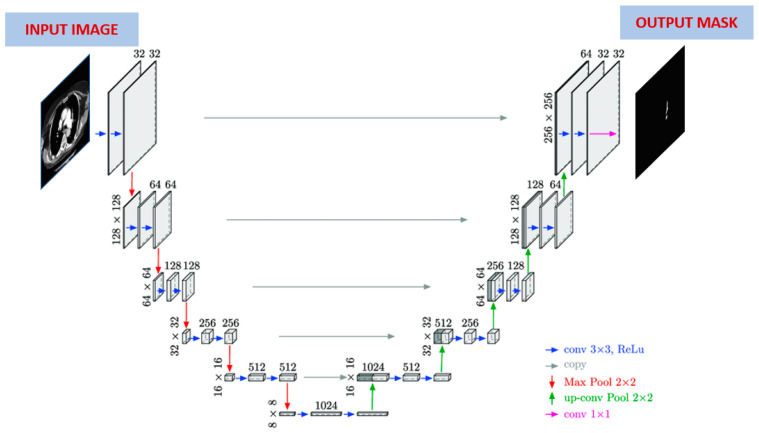
U-Net architecture [37].

The performance of the enhanced Mask R-CNN algorithm was compared with that of the U-Net algorithm. U-Net is a widely used deep learning model designed for image segmentation tasks, especially in medical imaging. It consists of an encoder, which extracts features from input images using convolutional layers, and a decoder, which reconstructs segmented images based on these features. U-Net is known for its ability to produce detailed segmentation results close to the original resolution of the image, making it effective for precise organ or lesion identification in medical images. The architecture of U-Net is shown in Figure 4. As its shape resembles the letter U, it was named after it.

There are two parts in this architecture. The first part is known as contraction (encoding) and the second part as expansion (decoding). The encoding part uses a traditional CNN architecture, in which the image size is slowly reduced using convolutional and maximum pooling layers, where the former consist of 3 × 3 filters and the latter consist of 2 × 2 filters. In the decoding part, which is completely symmetric with respect to the first part, the feature map is enlarged step-by-step through deconvolution towards the actual size of the image. Finally, each convolutional layer in the architecture is followed by an activation layer.

### 2.6. Evaluation Metrics

**Figure 5:**
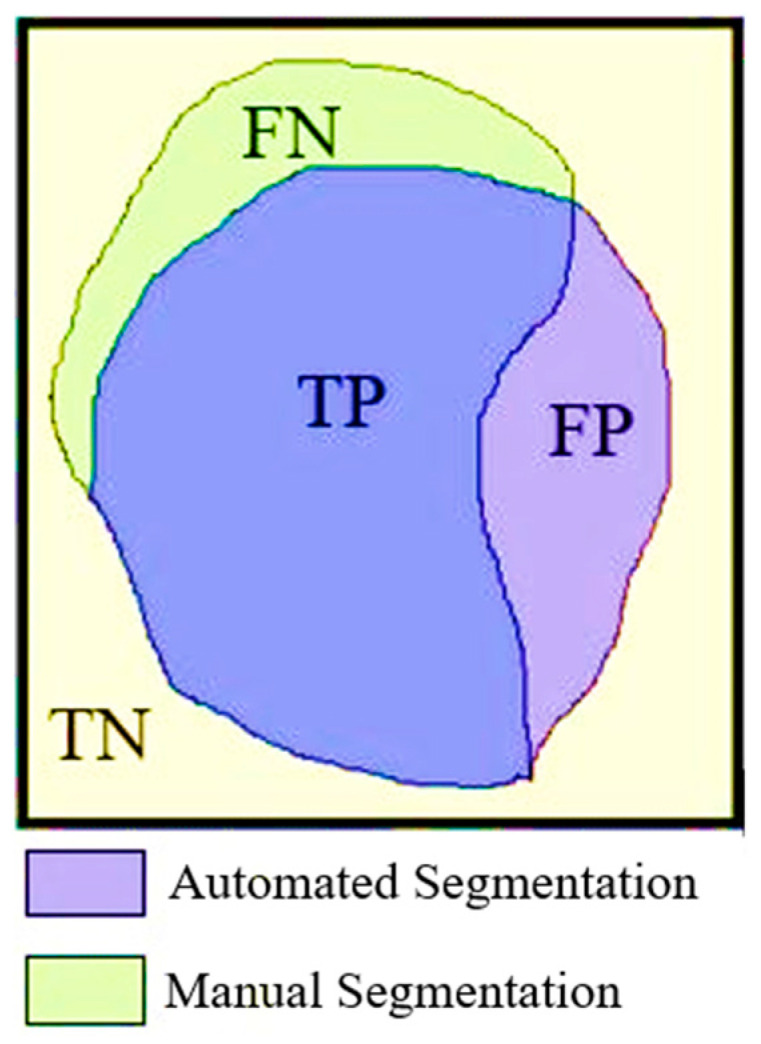
Representative overlap of automatic and manual segmentation when calculating similarity index performances.

Sensitivity, specificity, accuracy, and the Dice and Jaccard indices were used to assess the performance of Mask R-CNN in detecting pulmonary embolism. Sensitivity refers to the proportion of PE within the detected pixels; low sensitivity values indicate that true lesions are not adequately detected, while high sensitivity values indicate that the system is able to detect a high proportion of the regions that are recognized as lesions. As evaluating different criteria together gives more accurate results, the following were considered (see Figure 5): the true positive (TP), true negative (TN), false positive (FP), and false negative (FN) areas of the pixel groups obtained through automatic and manual segmentation. The equations for the performance parameters used are given as shown in Equations (3)–(7) below.

(3) $$Sensitivity=\frac{TP}{TP+FN},$$

(4) $$Specifity=\frac{TN}{TN+FP},$$

(5) $$Accuracy=\frac{TP+TN}{TP+TN+FP+FN},$$

(6) $$Dice=\frac{2xTP}{FP+2xTP+FN},$$

(7) $$Jaccard=\frac{TP}{FP+TP+FN}.$$

## 3. Results and Discussion

**Figure 6:**
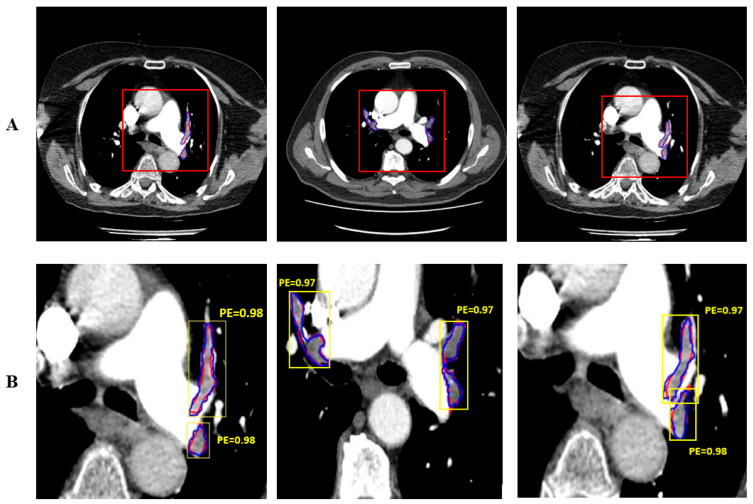
The automatic and manual segmentation of PE and detection performance: the (**A**) original images and (**B**) enlarged images.

Thorax CT images obtained from 50 patients with pulmonary embolism were used in this study, and images containing a total of 430 pulmonary embolisms were created by extracting sections of these images containing pulmonary embolisms. This study was carried out in four stages: data augmentation, pre-processing, PE segmentation, and performance evaluation. The pre-processed images were used as inputs to Mask R-CNN. The images of 36 patients (1505) were used for training, and those of 14 patients (645) were used for testing. Feature extraction for PE detection was performed with the ResNet50 convolutional neural network pre-trained on the COCO data set. Both hold-out validation (with a training/testing ratio of 70%:30%) and 10-fold cross-validation were used for performance evaluation. Figure 6 shows PEs automatically segmented by the proposed system (shown in blue) and those manually segmented by the expert doctor (shown in red). Mask R-CNN performed both detection and segmentation; in the figure, the detection of PEs is shown with yellow bounding boxes.

**Figure 7:**
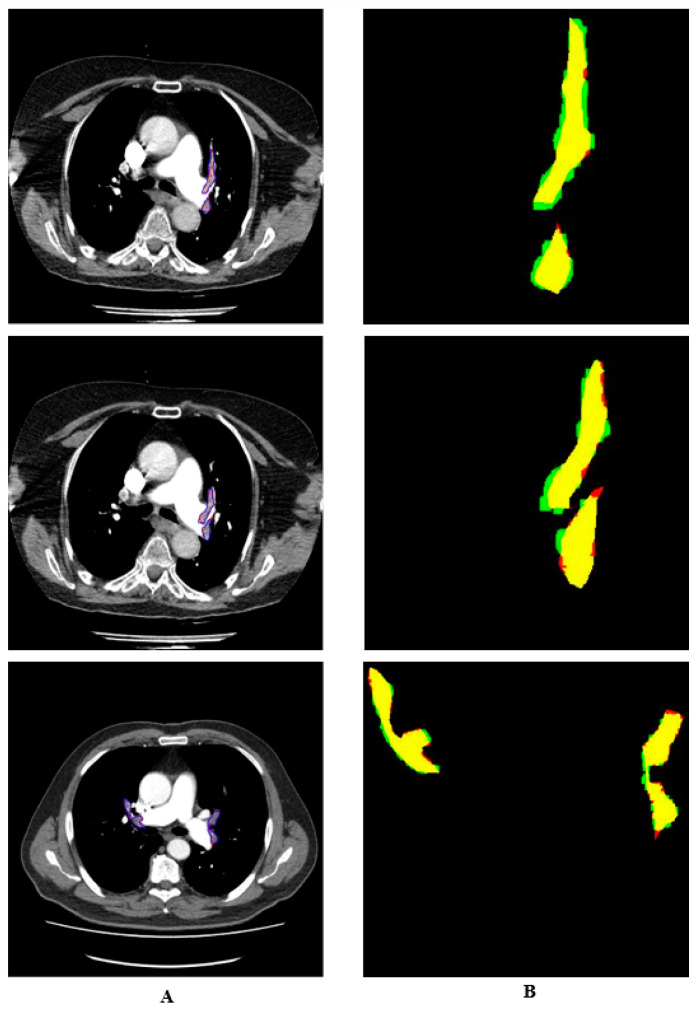
Drawings of detected PE (**A**) Colored drawings of manually and automatically segmented PEs (**B**).

The manual and automatic segmentation results are shown in different colors in Figure 7. The regions identified by the expert but not detected by the system are shown in red, while those detected as PEs by the system but not by the doctor are shown in green. The pixels belonging to PEs detected in both ways are shown in yellow. It can be seen that the proposed system could detect PEs with high performances.

**Figure 8:**
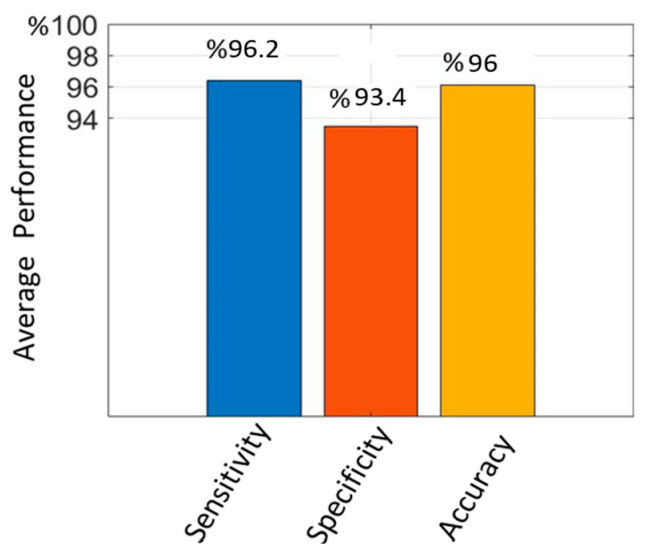
Average PE detection performance values following hold-out validation.

The average sensitivity, specificity, and accuracy values obtained on the test data with the proposed method following hold-out validation are given in Figure 8. The average sensitivity was 96.2%, the specificity was 93.4%, and the accuracy was 96%. It can be seen that the proposed enhanced Mask R-CNN presented a high performance in terms of PE detection.

**Figure 9:**
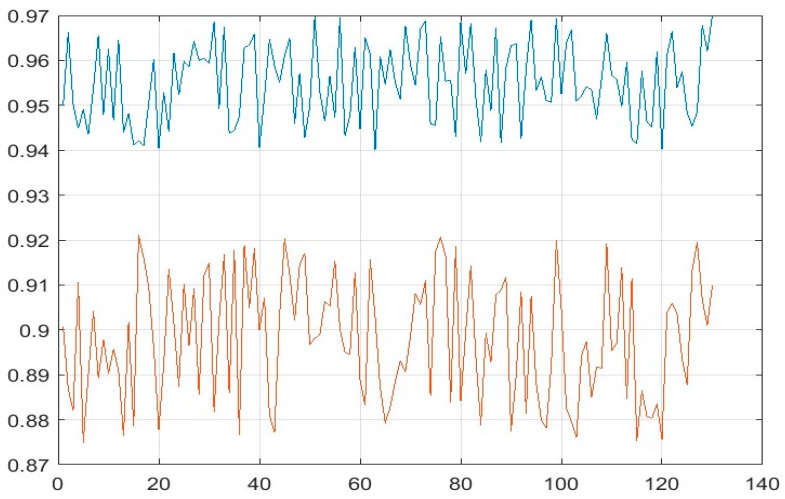
Dice and Jaccard similarity index values for test images.

The Dice and Jaccard similarity indices were also calculated in order to determine the similarity between automatic and manual PE detection. The minimum and maximum values were found to be 0.95 and 0.97 for the Dice index, respectively, and 0.88 and 0.91 for the Jaccard index, respectively. Figure 9 shows the Dice and Jaccard values obtained on the test images. The mean values obtained on the test data, as shown in Table 1, were 0.96 and 0.90 for the Dice and Jaccard similarity indices, respectively. The performance of the proposed method was tested using both hold-out validation and 10-fold cross-validation. Table 1 illustrates the superior performance of the enhanced Mask R-CNN, compared with Mask R-CNN, in terms of both Dice and Jaccard scores. The significant improvement observed, particularly in the 10-fold cross-validation results, suggests that the enhanced Mask R-CNN model possesses greater generalizability and reliability.

The PE detection performances of the enhanced Mask R-CNN, U-Net, and classical Mask R-CNN models were compared, as shown in Table 2. The results indicate that the enhanced Mask R-CNN provided higher sensitivity, specificity, and accuracy values than the classical Mask R-CNN and U-Net models. In particular, the fact that the sensitivity value obtained using the enhanced Mask R-CNN model was higher indicates that it has a higher success rate in detecting the lesion pixels compared with the other two methods.

The accuracy values obtained under different *λ* values are given in Table 3, where *λ*_1_ is the coefficient of the loss function of the bounding box (*L_box_*) and *λ*_2_ is the coefficient of the loss function of the mask (*L_mask_*). The results in the table indicate that varying the *λ*_1_ and *λ*_2_ values together impacted the model’s accuracy performance differently. For instance, when *λ*_1_ was held constant at 1 and *λ*_2_ was reduced from 1 to 0.8, both the hold-out and 10-fold CV accuracies generally increased. This illustrates that balancing the regularization coefficients can improve the model’s performance. Specifically, the optimal performance observed in this study was obtained when *λ*_1_ = 0.9 and *λ*_2_ = 0.8. In particular, the case where *λ*_1_ = 1 and *λ*_2_ = 1 corresponds to the standard configuration of the classical Mask R-CNN.

## 4. Conclusions

The effectiveness of a deep learning model during its training process is determined by the choice of its loss function, which is an especially critical component in models, such as Mask R-CNN, used for segmentation tasks to achieve accurate pixel-level predictions. The integration of a meticulously chosen loss function into the Mask R-CNN architecture significantly enhanced the model’s performance when compared with the traditional Mask R-CNN and U-Net models. This improvement was exemplified by the efficacy results reported in this study, where the enhanced Mask R-CNN achieved high sensitivity (96.2%), specificity (93.4%), and accuracy (96%) for the detection of pulmonary embolism within segmental arteries in computed tomography (CT) images. The results of this study underscore the pivotal role of the loss function in optimizing the model’s capacity to precisely identify and segment pulmonary embolisms. Enhancements and refinements in CNN-based models can significantly improve outcomes in object detection and segmentation tasks related to CT images. We hope that this study will inspire research into new loss functions that are particularly effective for specific tasks or applications.

## Figures and Tables

**Table 1:** Comparison of segmentation performances of Mask R-CNN and enhanced Mask R-CNN.

	Hold-Out Val.		10-Fold CV.	
	Dice	Jaccard	Dice	Jaccard
MASK R-CNN	0.91	0.87	0.87	0.82
ENHANCED MASK R-CNN	0.95	0.89	0.94	0.84

**Table 2:** Comparison of the enhanced Mask R-CNN with U-Net and Mask R-CNN.

	Hold-Out Val.			10-Fold CV		
Method	Sen. (%)	Spec. (%)	Acc. (%)	Sen. (%)	Spec. (%)	Acc. (%)
U-Net	90.7	87.8	90.5	88.2	86.7	88.6
Mask R-CNN	93.1	90	94.9	92	87.3	91.8
Enhanced Mask R-CNN	96.2	93.4	96	95.9	90	95.4

**Table 3:** Accuracy values for different λ_1_ and λ_2_ values.

$\lambda_{1}$	$\lambda_{2}$	Hold-Out Accuracy (%)	10-Fold CV Accuracy (%)
1	1	94.9	91.8
1	0.9	95	93.7
1	0.8	95.1	94.2
1	0.7	93.4	93.5
0.9	0.9	95.5	94.2
0.9	0.8	96	95.4
0.8	0.9	93.2	92.7
0.8	0.8	92.8	91.1

## Data Availability

The original contributions presented in the study are included in the article, further inquiries can be directed to the corresponding author/s.

## References

[1] Pforte A. (2004). Epidemiology, diagnosis, and therapy of pulmonary embolism. Eur. J. Med. Res..

[2] Goldhaber S.Z., Bounameaux H.J.T.L. (2012). Pulmonary embolism and deep vein thrombosis. Lancet.

[3] Pena E., Dennie C. (2012). Acute and chronic pulmonary embolism: An in-depth review for radiologists through the use of frequently asked questions. Seminars in Ultrasound, CT and MRI.

[4] Sadigh G., Kelly A.M., Cronin P. (2011). Challenges, controversies, and hot topics in pulmonary embolism imaging. Am. J. Roentgenol..

[5] Kumamaru K.K., Hunsaker A.R., Kumamaru H., George E., Bedayat A., Rybicki F.J. (2013). Correlation between early direct communication of positive CT pulmonary angiography findings and enhanced clinical outcomes. Chest.

[6] Leung A.N., Bull T.M., Jaeschke R., Lockwood C.J., Boiselle P.M., Hurwitz L.M., James A.H., McCullough L.B., Menda Y., Paidas M.J. (2011). An official American Thoracic Society/Society of Thoracic Radiology clinical practice guideline: Evaluation of suspected pulmonary embolism in pregnancy. Am. J. Respir. Crit. Care Med..

[7] Doubeni C.A., Corley D.A., Quinn V.P., Jensen C.D., Zauber A.G., Goodman M., Levin T.R. (2021). Effect of Organized Colorectal Cancer Screening on Mortality in a Large, Community-Based Population. JAMA Netw. Open.

[8] Torbicki A.T., Van Beek E.J.R., Charbonnier B., Meyer G., Morpurgo M., Palla A., Perrier A., Galie N., Gorge G., Herold C. (2014). ESC Guidelines on the diagnosis and management of acute pulmonary embolism. Eur. Heart J..

[9] Hartmann I.J.C., Prokop M. (2002). Spiral CT in the diagnosis of acute pulmonary embolism. Kontraste.

[10] Stein P.D., Fowler S.E., Goodman L.R., Gottschalk A., Hales C.A., Hull R.D., Leeper K.V., Popovich J., Quinn D.A., Sos T.A. (2006). Multidetector computed tomography for acute pulmonary embolism. N. Engl. J. Med..

[11] Yavas U.S., Calisir C., Ozkan I.R. (2008). The interobserver agreement between residents and experienced radiologists for detecting pulmonary embolism and DVT with using CT pulmonary angiography and indirect CT venography. Korean J. Radiol..

[12] Rufener S.L., Patel S., Kazerooni E.A., Schipper M., Kelly A.M. (2008). Comparison of on-call radiology resident and faculty interpretation of 4-and 16-row multidetector CT pulmonary angiography with indirect CT venography. Acad. Radiol..

[13] Joshi R., Wu K., Kaicker J., Choudur H. (2014). Reliability of on-call radiology residents’ interpretation of 64-slice CT pulmonary angiography for the detection of pulmonary embolism. Acta Radiol..

[14] Kline T., Kline T.J.R. (1992). Radiologists, communication, and Resolution 5: A medicolegal issue. Radiology.

[15] Qanadli S.D., El Hajjam M., Vieillard-Baron A., Joseph T., Mesurolle B., Oliva V.L., Barré O., Bruckert F., Dubourg O., Lacombe P. (2001). New CT index to quantify arterial obstruction in pulmonary embolism: Comparison with angiographic index and echocardiography. Am. J. Roentgenol..

[16] Mastora I., Remy-Jardin M., Masson P., Galland E., Delannoy V., Bauchart J.-J., Remy J. (2003). Severity of acute pulmonary embolism: Evaluation of a new spiral CT angiographic score in correlation with echocardiographic data. Eur. Radiol..

[17] Shiina Y., Funabashi N., Fujikawa A., Lee K., Sekine T., Uehara M., Mikami Y., Tanabe N., Kuriyama T., Komuro I. (2008). Quantitative evaluation of chronic pulmonary thromboemboli by multislice CT compared with pulsed Tissue Doppler Imaging and its relationship with brain natriuretic peptide. Int. J. Cardiol..

[18] Patil S., Henry J.W., Rubenfire M., Stein P.D. (1993). Neural network in the clinical diagnosis of acute pulmonary embolism. Chest.

[19] Tourassi G.D., Floyd C.E., Sostman H.D., Coleman R.E. (1995). Artificial neural network for diagnosis of acute pulmonary embolism: Effect of case and observer selection. Radiology.

[20] Scott J., Palmer E.J.R. (1993). Neural network analysis of ventilation-perfusion lung scans. Radiology.

[21] Wittenberg R., Berger F.H., Peters J.F., Weber M., van Hoorn F., Beenen L.F.M., van Doorn M.M.A.C., van Schuppen J., Zijlstra I.A.J., Prokop M. (2012). Acute pulmonary embolism: Effect of a computer-assisted detection prototype on diagnosis—An observer study. Radiology.

[22] Kligerman S.J., Lahiji K., Galvin J.R., Stokum C., White C.S. (2014). Missed pulmonary emboli on CT angiography: Assessment with pulmonary embolism-computer-aided detection. Am. J. Roentgenol..

[23] Bettmann M.A., White R.D., Woodard P.K., Abbara S., Atalay M.K., Dorbala S., Haramati L.B., Hendel R.C., Martin E.T., Ryan T. (2012). ACR Appropriateness Criteria^®^ acute chest pain—Suspected pulmonary embolism. J. Thorac. Imaging.

[24] Ozkan H., Tulum G., Osman O., Sahin S. (2017). Automatic detection of pulmonary embolism in CTA images using machine learning. Elektron. Ir Elektrotech..

[25] Hamet P., Tremblay J. (2017). Artificial intelligence in medicine. Metabolism.

[26] Liitjens G., Kooi T., Bejnordi B.E., Setio A.A.A., Ciompi F., Ghafoorian M., van der Laak J.A.W.M., van Ginneken B., Sánchez C.I. (2017). A survey on deep learning in medical image analysis. Med. Image Anal..

[27] Tao Q., Yan W., Wang Y., Paiman E.H., Shamonin D.P., Garg P., Plein S., Huang L., Xia L., Sramko M. (2019). Deep learning–based method for fully automatic quantification of left ventricle function from cine MR images: A multivendor, multicenter study. Radiology.

[28] Tajbakhsh N., Gotway M.B., Liang J. (2015). Computer-aided pulmonary embolism detection using a novel vessel-aligned multi-planar image representation and convolutional neural networks. International Conference on Medical Image Computing and Computer-Assisted Intervention, Proceedings of the 18th International Conference, Munich, Germany, 5–9 October 2015, Part II.

[29] Yang X., Lin Y., Su J., Wang X., Li X., Lin J., Cheng K.-T. (2019). A two-stage convolutional neural network for pulmonary embolism detection from CTPA images. IEEE Access.

[30] Pham A.-D., Névéol A., Lavergne T., Yasunaga D., Clément O., Meyer G., Morello R., Burgun A. (2014). Natural language processing of radiology reports for the detection of thromboembolic diseases and clinically relevant incidental findings. BMC Bioinform..

[31] Moore A.J.E., Wachsmann J., Chamarthy M.R., Panjikaran L., Tanabe Y., Rajiah P. (2018). Imaging of acute pulmonary embolism: An update. Cardiovasc. Diagn. Ther..

[32] Chen M.C., Ball R.L., Yang L., Moradzadeh N., Chapman B.E., Larson D.B., Langlotz C.P., Amrhein T.J., Lungren M.P. (2018). Deep learning to classify radiology free-text reports. Radiology.

[33] Grenier P.A., Ayobi A., Quenet S., Tassy M., Marx M., Chow D.S., Weinberg B.D., Chang P.D., Chaibi Y. (2023). Deep Learning-Based Algorithm for Automatic Detection of Pulmonary Embolism in Chest CT Angiograms. Diagnostics.

[34] Wu M., Li C., Yao Z. (2022). Deep Active Learning for Computer Vision Tasks: Methodologies, Applications, and Challenges. Appl. Sci..

[35] Khan M., Shah P.M., Khan I.A., Islam S.U., Ahmad Z., Khan F., Lee Y. (2023). IoMT EnabledComputer-Aided Diagnosis of Pulmonary Embolism from Computed Tomography Scans Using Deep Learning. Sensors.

[36] Vainio T., Mäkelä T., Arkko A., Savolainen S., Kangasniemi M. (2023). Leveraging open dataset and transfer learning for accurate recognition of chronic pulmonary embolism from CT angiogram maximum intensity projection images. Eur. Radiol. Exp..

[37] Ronneberger O., Fischer P., Brox T. (2015). U-Net: Convolutional Networks for Biomedical Image Segmentation. Medical Image Computing and Computer-Assisted Intervention–MICCAI 2015, Proceedings of the 18th International Conference, Munich, Germany, 5–9 October 2015.

[38] Ren S., He K., Girshick R., Sun J. Faster r-cnn: Towards real-time object detection with region proposal networks. Proceedings of the Advances in Neural Information Processing Systems 28 (NIPS 2015).

[39] He K., Gkioxari G., Dollár P., Girshick R. Mask r-cnn. Proceedings of the IEEE International Conference on Computer Vision.

[40] He K., Zhang X., Ren S., Sun J. Deep residual learning for image recognition. Proceedings of the IEEE Computer Society Conference on Computer Vision and Pattern Recognition (CVPR).

